# Effect of fertilizers on yield, phytochemical, and antioxidant properties of *Cucurbita moschata* fruits

**DOI:** 10.1002/fsn3.4294

**Published:** 2024-07-09

**Authors:** Boudjeka Guemkam Vanessa, Tchiazé Ifoué Alice, Djeukeu Asongni William, Demasse Mawamba Adelaide, Loé‐Etame Gisèle, Nina‐Nicoleta Condurache, Ștefania‐Adelina Milea, Mihaela Cotarlet, Dongho Dongmo Fabrice, Gouado Inocent, Gabriela Iordachescu

**Affiliations:** ^1^ Research Laboratory of Biotechnology, University Institute of Technology University of Douala Douala Cameroon; ^2^ Food Science and Nutrition Laboratory, Faculty of Sciences University of Douala Douala Cameroon; ^3^ Laboratory of Plant Biology, Faculty of Sciences University of Douala Douala Cameroon; ^4^ Department of Home Economic, Advanced Teacher's Training College for Technical Education University of Douala Douala Cameroon; ^5^ Department of Pharmacy, Faculty of Medicine and Pharmaceutical Sciences University of Douala Douala Cameroon; ^6^ Plant Biotechnology and Consumer Sciences Laboratory, Bioaliment Platform, Faculty of Food Engineering University of Dunarea de Jos Galati Romania

**Keywords:** antioxidant properties, fertilization, phytochemical composition, squashes farming, yield

## Abstract

The nutritional and functional properties of squashes are influenced by various factors, such as the stage of plant development, soil composition, and type of fertilizer. This study evaluates the impact of various organic fertilizers on *Cucurbita moschata* D. properties. For this purpose, squashes were fertilized using Ash at 10 kg/25 m^2^, bovine compost at 62.5 kg/25 m^2^, and a 1:1 mixture of ash and bovine compost. Negative control (without fertilizers) and positive control (NPK 20‐10‐10 at 2 kg/25 m^2^) were included. Post‐harvest, the yield, carotenoids, phenolic compounds, flavonoids, and antioxidant activities were assessed. Ash fertilizer resulted in the highest number of fruits per plant (2.20 ± 0.16). Regarding flavonoids, the bovine compost yielded the highest level (428.67 ± 2.62 mg/100 g of edible portion). The mixture of ash and bovine compost produced fruits with the highest content of total carotenoids, β‐carotene, lycopene, and total phenolic compounds (249.7 ± 3.68, 219.80 ± 3.41, 26.07 ± 0.41, and 575.00 ± 9.95 mg/100 g of edible portion, respectively). Moreover, carotenoids extracted from this mixture exhibited the highest inhibition of DPPH and ABTS free radicals (30.87 ± 0.65 and 47.32 ± 1.30%, respectively). This research suggests that the use of a mixture of ash and bovine compost could significantly enhance the yield of squashes and their phytochemical and antioxidant potential.

## Graphical abstract

This study explores the impact of different organic fertilizers on the yield and nutritional properties of *Cucurbita moschata*. It was found that a mixture of ash and bovine compost significantly enhanced the yield, phytochemical content, and antioxidant potential of the squashes, suggesting its potential as an effective organic fertilizer.

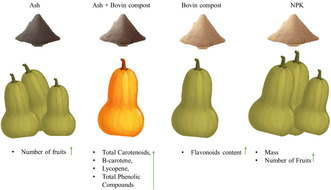

## 1 INTRODUCTION

Pumpkin, a member of the *Cucurbitaceae* family, is a large fruit that can be either round or oblong. It features a thick, orange or yellowish skin and a hollow interior filled with seeds and stringy flesh (Caili et al., 2006). This fruit is widely cultivated for its edible flesh, which is incorporated into a variety of dishes such as soups, pies, and baked goods. Among the six species of pumpkin, three (*Curcubita pepo* L., *Curcubita maxima* D., and *Curcubita moschata* D.) hold significant economic value and are cultivated globally (Sonu Sharma & Ramana Rao, 2013).

*Cucurbita moschata* is one of the most popular and widely consumed species of pumpkin. Studies have indicated that this species can aid in the prevention and control of certain diseases, such as high blood pressure, cardiovascular diseases, vitamin A deficiency (Demasse et al., 2009, 2021), obesity, and type II diabetes (Boudjeka et al., 2014). These health benefits are attributed to the presence of phytochemicals such as polysaccharides, carotenoids, vitamin C, phenolic compounds, and others in its pulp (Boudjeka et al., 2021; Demasse et al., 2009).

Despite being cultivated across all agro‐ecological zones of Cameroon (Demasse et al., 2021), the country's pumpkin production is relatively low (205,471 tons produced in 2019 compared to China's 8,427,676 tons). To boost crop yields, farmers often resort to various fertilizers. The application of organic manure is known to enhance soil physical properties and maintain soil organic matter (Adekiya et al., 2020; Nweke, 2020; Pso & Nweke, 2015). The use of poultry droppings, animal manure, sewage sludge, and municipal waste on agricultural land has been demonstrated to improve productivity (Ma et al., 2023).

Numerous studies have unveiled the effects of organic and chemical fertilizers on the nutritional and antioxidant contents of plant tissues. Génard et al. (2020) demonstrated that soil composition can influence the phytonutrient potential of cultivated plant species. Ibrahim et al. (2013) reported that the use of organic fertilizer boosts secondary metabolite production and antioxidant activity, such as total phenolics and DPPH radical scavenging of *Labisia pumila* B., compared to the use of chemical fertilizers. Additionally, the impact of fertilizers on the yield of vegetable crops such as ginger and red chili has been demonstrated (Adekiya et al., 2020; Wisnubroto et al., 2017). Muscolo et al. (2020) showed that the use of sulfur‐organic‐based fertilizers increased the production of bioactive organo‐sulfur compounds and antioxidants in red onion, depending on the type and concentration of sulfur‐organic‐based fertilizer used. These findings were corroborated by Matrella et al. (2022), who showed that the quantity and quality of bioactive compounds contained in the onion bulb can vary based on cultivation practices.

However, the use of fertilizers by pumpkin farmers in Cameroon is limited, and there is a dearth of studies on the effect of fertilizers on the phytochemical content of pumpkin fruit. The lack of studies on this domain is a significant gap in the literature. Understanding how different fertilization methods can influence the levels of these compounds in *Cucurbita moschata* could lead to improved cultivation practices that maximize both yield and nutritional value. This study was therefore designed to assess the effect of biological and chemical fertilizers on the yield, phytochemical composition (carotenoids and phenolic compound content), and antioxidant properties of *Cucurbita moschata*.

## 2 MATERIALS AND METHODS

### 2.1 Site description and experimental layout

The study was conducted in the agro‐ecological region of the Western Highlands, specifically within the Koung‐khi sub‐division and the Baa‐Bayangam locality. This area is characterized by a climate known as the “Cameroonian altitude”, which is marked by two distinct seasons of varying lengths: a dry season that spans from mid‐November to mid‐March and a rainy season that lasts from mid‐March to mid‐November. The region maintains a relatively cool average temperature of 19°C and receives substantial rainfall, ranging from 1500 to 2000 mm, following a single‐mode pattern. Prior to this study, the land was used for the cultivation of leafy vegetables.

The experimental procedure involved five distinct treatments:

1. Control group with no fertilizer application (T_0_)
2. Application of only ash at a rate of 10 kg/25 m^2^ (T_1_)
3. Application of only bovine compost at a rate of 62.5 kg/25 m^2^ (T_2_)
4. Combined application of 50% ash at 10 kg/25 m^2^ and 50% bovine compost at 62.5 kg/25 m^2^ (T_3_)
5. Application of NPK (20‐10‐10) fertilizer at 2 kg/25 m^2^ as a positive control (T_4_)

The treatments were devised in accordance with the nutritional requirements of pumpkins, as outlined by Björkman and Reiners (2014). The experimental setup adhered to a randomized complete block design, replicated thrice. Each block of the experiment covered an area of 25 m^2^. To prevent interference, plots were spaced 1 m apart, while a distance of 4 m was maintained between the blocks. This experiment was repeated at the identical location in 2022. Before initiating the experiment, the soil's physicochemical parameters were analyzed. Soil samples weighing 2 kg were randomly collected from 10 different points across the study area using steel coring tubes at a depth of 0–20 cm. These samples were combined, air‐dried at 35°C for 24 h, sieved, and then analyzed for particle size, pH, organic carbon, organic matter, total nitrogen (N), phosphorous (P), potassium (K), sodium (Na), calcium (Ca), and magnesium (Mg).

#### 2.1.1 Soil particle size determination

The process of determining soil particle size was accomplished through mechanical analysis. In this procedure, 10 g of soil was subjected to a treatment of 50 mL of hydrogen peroxide to remove organic matter. The decomposition of organic matter was accelerated by applying a gentle heat to a hot plate. The sand fraction was separated from the rest by sieving underwater using a sieve with a mesh size of 50 μm. The silt and clay fractions were gathered using a Robinson‐Köhn pipette. This was carried out after the soil was dispersed with sodium hexametaphosphate, and the resulting suspension, which comprised silts and clays, was agitated with a rotary stirrer. Upon calculating the proportions of the different textural fractions, the textural classes were identified using the Textural Triangle provided by the United States Department of Agriculture (USDA).

#### 2.1.2 Soil pH estimation

The evaluation of soil pH was performed using a CG822 model pH meter, outfitted with a combined pH electrode. Two pH variants, pH‐KCl and pH‐H_2_O, were assessed. The actual acidity, denoted by pH‐H_2_O, was ascertained in a soil–water suspension at a ratio of 1:2.5 (10 g of soil combined with 25 mL of distilled water). This measurement was recorded no less than 16 h after the preparation of the suspension.

#### 2.1.3 Organic carbon estimation

The quantification of organic carbon and organic matter was executed using the Walkley and Black method, as outlined by Pauwels et al. (1992). This technique involves the oxidation of organic carbon using potassium dichromate (K_2_Cr_2_O_7_) in a highly acidic environment (H_2_SO_4_). The excess K_2_Cr_2_O_7_ is then titrated back using ferrous sulfate (FeSO_4_.7H_2_O), which facilitates the calculation of the amount of dichromate that has been neutralized by the organic carbon. The completion of this process is marked by the color change of diphenylamine [(C_6_H_5_) 2NH_3_] from purple to green. The percentage of organic carbon (OC) was computed using the following formula

$$\%\mathrm{OC}=4\;\left(V_{0}-V\right)\times 100/V_{0}.T_{s}$$

V_0_ = Volume of FeSO_4_.7H_2_O added to the control; V = Volume of FeSO_4_.7H_2_O added to the sample; T_s_ = test sample (0.5 g of soil).

The organic matter (OM) content is taken from the relationship:

%OM=%CO×1724

Bearnaert and Bitondo's (1992) study provides a framework for interpreting the rate and quality of organic matter, as outlined below:

Organic matter rate: Very low: <1.0%; Low: 1.0%–2.0%; Medium: 2.0%–4.2%; High: 4.2%–6.0%; Very high: >6.0%.
Organic matter quality (C/N ratio): Very poor: >20; Poor: 14–20; Good: 10–14; Very good: <10.

#### 2.1.4 Total nitrogen estimation

The total nitrogen content of samples was assessed by the Kjeldahl method, as described by Pauwels et al. (1992). Approximately 10 g of soil was completely mineralized by heat treatment with a mixture of concentrated sulfuric acid and salicylic acid. The CuSO_4_ + S mixture serves as a catalyst. Mineralization was followed by steam distillation of nitrogen as NH_3_ after basifying the mineralized extract with NaOH. The distillate was collected in boric acid (H_3_BO_3_) and then titrated using sulfuric acid (0.01 N). The total nitrogen content was calculated using the following formula:

$$N\;\left(g/\mathrm{kg}\;\text{soil}\right)=14\;\left(V-V_{0}\right)\;t/T_{s}$$

where V = volume of H_2_SO_4_ added to the sample; V_0_ = volume of H_2_SO_4_ added to the control; T_s_ = soil test taken in grams (2 g); t = normality of H_2_SO_4_ = 0.01 N; N (%) = N (g/kg of soil) * 0.1.

The cut‐off point for nitrogen content was assessed according to Ilaco (1981). Very low total nitrogen: <0.050%; Low: 0.050%–0.125%; Medium: 0.125%–0.225%; High: 0.225%–0.300%, and Very high: >0.300%.

#### 2.1.5 Phosphorous estimation

The Bray method (Bray & Kurtz, 1945) was employed to determine the available phosphorus. Subsequently, the extracted phosphorus was quantified using spectrophotometry with molybdenum blue, utilizing a molecular absorption spectrophotometer at a wavelength of 665 nm. The evaluation of the available phosphorus was conducted in accordance with Ilaco (1981). The assimilable phosphorus levels were classified as follows: Very Low: less than 7 ppm; Low: between 7 and 16 ppm; Medium: between 16 and 46 ppm; and High: greater than 46 ppm.

#### 2.1.6 Other mineral estimations

Potassium, natrium, calcium, and magnesium contents were determined by atomic absorption spectrometry.

### 2.2 Fertilizer procurement

The fertilizers used for the study are traditional organic fertilizers, in particular, ash, bovine compost and the ash + compost mixture. NPK (20‐10‐10) fertilizer was purchased from a local producer.

#### 2.2.1 Ash production

Ash was obtained as follows: The untreated eucalyptus branches and stems, devoid of any plastic waste, were calcined by combustion in an oven at 550°C for 4 h. After cooling the raw ash obtained, it was sieved using a sieve with a 2 μm diameter. The fine ash obtained was then stored in sealed plastic bags for future use.

#### 2.2.2 Bovine compost preparation

Bovine compost was prepared essentially from the urine and feces of cattle mixed with wood chips, water, and shredded plant residues in a ratio of 1:4. The materials were piled up in successive layers. Stems of plants are cut into pieces, followed by the excrement of urine and bovine feces, and water is added to the optimum. When forming the pile, a few bamboo stems are pushed in to facilitate ventilation. When the pile is ready, it is surrounded by a layer of mud 3 cm thick. The bamboo stems were removed on the second day of composting, leaving holes that allow aeration. After 5 days, the temperature rises to 70°C and the holes are closed in turn. The first turnaround was done after 3 weeks. The humidity of the pile was adjusted with water and cattle dung, and the overturned pile was again sealed from the air with mud. The compost was used after 3 months of growing the squash.

Before the start of the experiment, fertilizer samples were analyzed for organic carbon, organic matter (Bearnaert & Bitondo, 1992), N, P, K, Ca, Mg, and pH following the methods described above (Bray & Kurtz, 1945; Ilaco, 1981).

### 2.3 Crop and fertilizer application

The fields underwent plowing from March to April 2022. Two weeks prior to sowing, biological fertilizers were applied to the soil as base manure, followed by maintenance manure at intervals of 30, 60, and 90 days post‐sowing. The fertilizers were spread by burying them 10 to 20 cm deep. In May 2022, pumpkin seeds were sown 2 cm deep. For each hole, two pumpkin seeds (*C. moschata* of the Butternut variety) were planted with the tip facing downward. A spacing of 1.60 m between rows and 70 cm within rows was maintained, achieving a density of one plant per square meter. Hand weeding was performed at monthly intervals throughout the experiment. The plants were left unpruned during cultivation. Crop maintenance, which included bi‐monthly weeding and daily morning watering, was carried out until harvest, following the modified method of Adekiya et al. (2020).

### 2.4 Harvest and determination of yield parameters

The harvest took place 5 months later, in September, when the fruits reached maturity. Therefore, maturity was assessed by the drying of the leaves and the peduncle connecting the fruit to the stem, as well as the bright and frank color of the fruits. The harvest was carried out manually, and the fruits were allowed to dry for 48 h in the field. The fruits were harvested by eliminating those located at the ends of the plots. The parameters for evaluating the crop yield are the mass and the number of fruits per plant harvested (Thepsilvisut et al., 2022).

#### 2.4.1 Weight determination

The weight of fruits was measured at harvest using a balance (Pso & Nweke, 2015) (HEINNER HKS‐5SL, 0.1 g).

#### 2.4.2 Number of fruit estimations

The number of fruits per plant was determined at harvest by manual counting (Pso & Nweke, 2015).

### 2.5 Evaluation of the effect of fertilizers on the phytochemical composition and antioxidant potential of pumpkin fruit

After harvesting, pumpkins were weighed, and the water and dry matter contents were determined. These fruits were peeled, and the pulp was collected for phytochemical analyses. The pulp samples were finely ground, and a test sample of 2.5 g per sample was weighed. Several analyses have been carried out on these samples, including quantification of carotenoids, phenolic compounds, polysaccharides, and the evaluation of antioxidant potential.

#### 2.5.1 Determination of carotenoid content

The carotenoids were extracted by ultrasonication in an ultrasonic generator with 300 W power and 40 kHz frequency, according to the modified method of Lianfu and zelong (2008) using a mixture of hexane–acetone solvent (3:1). In a 20‐mL test tube, 2.5 g of pumpkin pulp were introduced, and 10 mL of solvent were added to it. The whole mixture was brought to ultrasonication for 40 min at 20°C. The crude extract obtained was recovered and then centrifuged for 15 min at 42.16 *g* and at 10°C. The supernatant was concentrated in a vacuum using a vacuum concentrator. The dry extracts obtained were subsequently used to determine the content of total carotenoids, β‐carotene, and lycopene in each sample of squash pulp. Carotenoid extraction was carried out until the squash pulp samples became discolored. After extraction, carotenoids were quantified by spectrophotometry (Bailey, 2015).

#### 2.5.2 Determination of phenolic content

The total phenolic compounds were extracted by ultrasonication using the ethanol–water solvent mixture (70:30). In a 10‐mL test tube, 1 g of squash pulp was introduced, and 5 mL of solvent were added to it. The whole was brought to ultra‐sonication for 40 min at 20°C. The crude extract obtained was recovered and then centrifuged for 15 min at 42.16 *g* at a temperature of 10°C. The supernatant was concentrated in vacuum using a vacuum concentrator. The dry extracts obtained were subsequently used to determine the content of total phenolic compounds and flavonoids in each sample of squash pulp. Phenolic compounds and flavonoids were subsequently quantified by spectrophotometry using the method described by Dewanto et al. (2002).

#### 2.5.3 Determination of polysaccharide content and evaluation of antioxidant activities

Total digestible polysaccharides were determined by spectrophotometry according to the method of (Dubois et al., 1956). The evaluation of the antioxidant activities was done by trapping the free radicals DPPH and ABTS, according to the modified method of Re et al. (1999).

### 2.6 Statistical analysis

EXCEL, GRAPHPAD PRISM, and MINITAB software were used for data processing. The average, standard deviations, ordered two‐dimensional analysis of variance, and weighted percentages were compared between different groups at the 5% significance level using the Fisher, Kruska–Wallis, Bonferroni, and Duncan tests.

## 3 RESULTS AND DISCUSSION

### 3.1 Soil and fertilizer composition

Table 1 shows the results of the physicochemical analyses of the soil for the crop. These results show that the soil consists of 75% sand, 5% silt, and 20% clay. Meanwhile, these results show that the soil has a pH of 4.6. The results of organic matter analyses show that the carbon content is 4.1%, the total organic matter content is 7.07%, the total nitrogen content is 0.15%, and the C/N ratio is 27. The results of the analysis of free cations like calcium, magnesium, potassium, and sodium are, respectively, 3.52; 1.12; 0.14, and 0.02 meq/100 g of DM. The sum of the exchangeable bases is 4.8 meq/100 g of DM, while the phosphorus content is 0.4 mg/kg of soil.

**TABLE 1:** Physicochemical composition of the soil.

Parameters			Values
Granulometry (%)	Sand	*Fine*	70.0
		*Coarse*	5.0
	Silt	*Fine*	4.0
		*Coarse*	1.0
	Clay		20.0
Soil reaction	pH‐water		4.6
	pH‐KCl		4.0
Organic material	Organic carbon (%)		4.1
	Organic matter (%)		7.07
	Total nitrogen (g/kg)		1.51
	C/N Ratio		27.0
Exchangeable cations (meq/100 g)	Calcium		3.52
	Magnesium		1.12
	Potassium		0.14
	Sodium		0.02
	Sum of bases		4.80
Phosphorus	Bray II (mg/kg)		0.4

Table 2 shows the results of the analysis of ash, bovine compost, and NPK fertilizer used for soil fertilization. Chemical characterization studies show variability in the composition of ash, bovine compost, and NPK used for soil fertilization. The results show that ash is rich in mineral elements, including potassium (65%), phosphorus (3.2%), calcium (3.1%), and magnesium (2.8%). Iron and manganese accumulate a percentage of 3%. The other elements, such as chlorine, sodium, and sulfur, are present in trace amounts, respectively, in proportions of 0.002%, 0.03%, and 0.033%. Bovine compost is characterized by high contents of organic matter (35.45%) and organic carbon (12.9%). The mineralization of the nitrogen contained in the compost used is illustrated by the C/N ratio, which has a value of 15. Bovine compost and ash have pH values of 7.05 and 10.1, respectively. NPK is essentially rich in mineral elements, including nitrogen (20%), phosphorus (10%), and potassium (10%).

**TABLE 2:** Physico‐chemical composition of fertilizers.

Parameters	Fertilizers		
	Ash	Bovine compost	NPK
SiO_2_ (%)	0.29	—	—
P (%)	3.20	0.65	10
K (%)	65.00	1.40	10
Ca (%)	3.10	—	—
Mg (%)	2.80	—	—
Na (%)	0.03	—	—
S (%)	0.033	—	—
Fe (%)	1.53	—	—
Mn	1.45	—	—
Cl (%)	0.002	—	—
N (%)	—	0.86	20
Organic carbon (%)	—	12.90	—
Organic matter (%)	—	35.45	—
C/N Ratio	—	15.00	—
pH	10.2	7.05	9.1

### 3.2 Effect of fertilizers on crop yield

Table 3 shows the evolution of the masses, numbers per plant, and water contents of pumpkins according to the fertilizers. Squashes fertilized with T_2_ (bovine compost) and T_4_ (NPK) produced the fruits with the largest masses. The use of compost alone or in combination with ash and the mineral NPK has improved the growth of squash compared to the negative control. These results are due to the favorable action of nitrogen contained in bovine compost, as well as in chemical fertilizer, on soil fertility (Jalali & Ranjbar, 2009). Indeed, the vegetative growth of plants is positively correlated with the absorption of nutrients, in particular nitrogen (50%–60% of its nitrogen content would be available to the plant), which plays an important role in increasing the index and leaf production as well as photosynthetic activity (Eleiwa & Mohamed, 2012). This provides a justification for the good growth of the squash plants observed following the application of fertilizers consisting of bovine compost. In addition, the applications of the organic fertilizer compound T_3_ (compost + ash) are more effective on the growth of squash plants than those of organic fertilizers T_2_ (compost). This would be due to the high availability of macroelements such as nitrogen and microelements such as magnesium contained in these organic fertilizers, which have a synergic action on the growth of squash plants. According to Choudhary et al. (2004) and El‐Magd and Abou‐Hussein (2008), organic matter improves growth by lowering the pH of the rhizosphere, which results in better solubilization of nutrients and high availability for plants. In addition, organic matter, which is an important source of mineral elements (N, P, K) necessary for the good development of the plant, particularly improves the structure of the soil and its capacity for water retention (Suganya & Sivasamy, 2006). Through the effects due to the T_3_ (compost + ash) treatment, it seems that the assimilation of nitrogen by plants is favored by the strong presence of potassium and phosphorus in the ash. According to Leikam et al. (1983), adequate nutrition of phosphorus and potassium can increase the growth response of the crop to nitrogen.

**TABLE 3:** Evolution of the masses, numbers per plant, and water contents of pumpkins according to the fertilizers used.

	Treatment					
	NPK	Compost	Ash	Compost + ash	No fertilizers	*p*
Mass (kg)	2.38^a^ ± 0.36	1.99^b^ ± 0.22	1.2^c^ ± 0.07	1.56^d^ ± 0.12	1.08^e^ ± 0.08	.0001
Number	2.30^a^ ± 0.16	1.65^b^ ± 0.11	2.20^a^ ± 0.16	2.00^a^ ± 0.13	0.950^c^ ± 0.14	.0001
Water content	85.55^a^ ± 0.30	93.26^b^ ± 0.38	87.57^c^ ± 0.30	88.06^c^ ± 0.28	88.73^c^ ± 0.64	.01

*Note*: Means with the same letter in each row are not significantly different at the 5% level of probability using the Kruskal–Wallis test.

The plants fertilized with T_1_ (ash) and T_3_ (compost + ash) treatments produced the highest number of fruits similar to the mineral NPK. The plants fertilized with T_2_ and T_3_ produced fruits having masses greater than T_0_ (negative control). TT This could be due to the additional effect of the high potassium contents in ash to that of the nitrogen contained in the compost, would have been decisive for a high yield in number and mass of fruit fertilized by T2 and T3 (Lester et al., 2005; Quaggio et al., 2011). In addition, the study has shown that T_2_ and T_3_ treatments have properties comparable to those of T_4_ (positive control) and are likely to replace the latter.

### 3.3 Effect of fertilizers on phytochemical and antioxidant potential

#### 3.3.1 Effect on phytochemical content

##### Effect on carotenoid content

**FIGURE 1:**
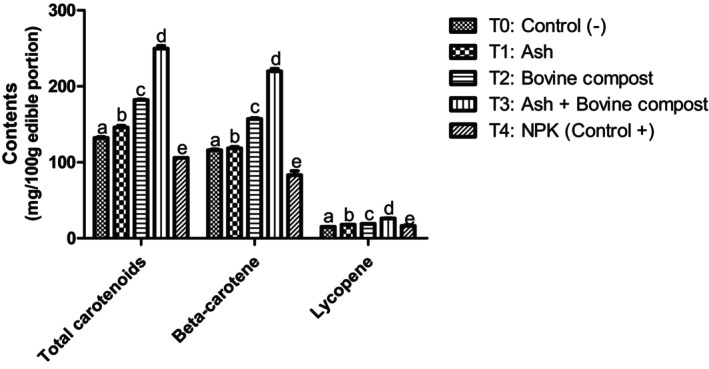
Total carotenoid, β‐carotene, and lycopene content in pumpkin samples. Means with the same letter in each diagram are not significantly different at the 5% level of probability using the Bonferroni test.

The application of fertilizers, as depicted in Figure 1, had a significant (*p* < .05) impact on the total carotenoids, β‐carotene, and lycopene content in squash fruits. Treatments with biological fertilizers, specifically T1, T2, and T3, resulted in fruits with the highest total carotenoids (145.90 ± 2.25, 182.10 ± 0.76, and 249.7 ± 3.68 mg/100 g edible portion, respectively), β‐carotene (118.5 ± 1.86, 157.1 ± 1.31, and 219.8 ± 3.41 mg/100 g edible portion, respectively), and lycopene contents (17.85 ± 0.21, 19.05 ± 0.07, and 26.07 ± 0.41 mg/100 g edible portion, respectively) compared to T4 (positive control). These results could be attributed to the high availability of macroelements such as nitrogen and microelements like magnesium and potassium contained in these organic composite fertilizers. According to Choudhary et al. (2004) and El‐Magd and Abou‐Hussein (2008), organic matter enhances growth by reducing the pH of the rhizosphere, leading to improved solubilization of nutrients and increased availability for plants. This, in turn, boosts the biosynthesis of organic molecules such as carbohydrates in plants.

**FIGURE 2:**
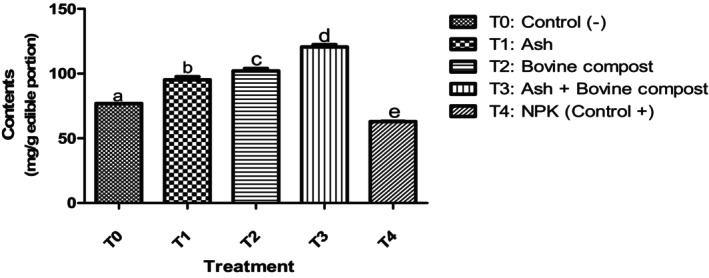
Total carbohydrates content in squash fruits. Means with the same letter in each diagram are not significantly different at the 5% level of probability using the Kruskal–Wallis test.

So these results could also be explained by the high levels of total carbohydrates in the squashes obtained with T_3_ treatment (Figure 2). Indeed, carbohydrates are the substrates used during the biosynthesis of carotenoids by the biosynthesis pathway of deoxyxylulose phosphate. The biosynthesis of carotenoids derives from the general biosynthesis of isoprenoids; the branching of these pathways takes place at the phytoene level. The universal precursor of isoprenoids is pyrophosphate isopentenyl (PPI), which is formed by the deoxyxylulose phosphate pathway in photosynthetic plants. In this way, the precursors of PPI are glyceraldehyde 3‐phosphate and pyruvate, resulting from the carbohydrate catabolism.

##### Effect on phenolic compound content

**FIGURE 3:**
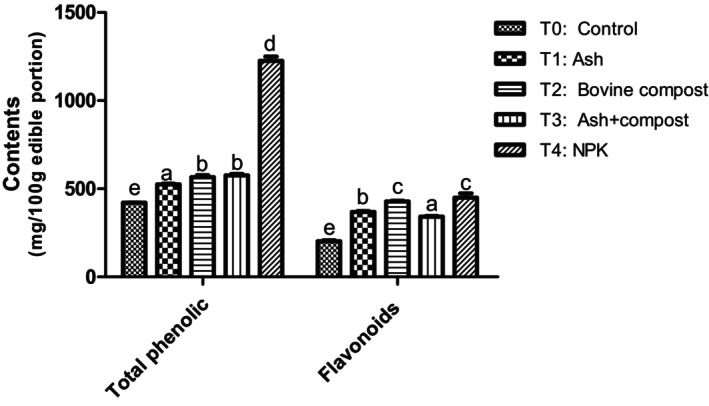
Total phenolic and flavonoid content in pumpkin samples. Means with the same letter in each diagram are not significantly different at the 5% level of probability using Bonferroni test.

Total phenolic and flavonoid compounds in squash fruits were also significantly influenced by fertilizers in comparison to the negative and positive controls (Figure 3).

According to biological fertilizers, T_2_ produced fruits with the highest total flavonoid contents in order of 428.67 ± 2.62 mg/100 g of edible portion, compared to T_1_ (368.70 ± 3.84 mg/100 g of edible portion) and T_3_ (341.63 ± 4.39 mg/100 g of edible portion). The use of compost alone (T_2_) or in combination with ash (T_3_) showed significantly (*p* < .05) higher contents of total phenolic compounds compared to the negative control (T_0_). In fact, organic nitrogen is the major component of compost, and therefore the supply of compost leads to a lesser accumulation of nitrate absorbable by the plant for the synthesis of secondary metabolites. In addition, the ashes from the ignition of wood and branches of wood are composed mainly of phosphorus and potassium oxide (P_2_O_5_, K_2_O) and some trace elements like magnesium, calcium, and manganese. They are very poor, almost devoid of nitrogen elements with a C/N ratio greater than 20; consequently, the availability of mineral nitrogen directly absorbable by the plant has been reduced, and hence the observation of this low total phenolic content. These are in accordance with those obtained by Premuzic et al. (2002), who showed that the supply of compost leads to less accumulation of nitrate but greater accumulation of vitamin C. Premuzic et al. (2001) also found similar results on the cherry tomato. In fact, the content of vitamin C was more important for an organic fertilization (compost) or a fertilization combining mineral and organic contributions. The highest level of total phenolic and total flavonoid contents observed in squash fruits fertilized with positive control (T_4_) is due to the presence of mineral nitrogen, directly available to the plant in the form of nitrate and ammonium. Thus, providing the accumulation of organic matter, in particular sugars from the pentose cycle and the Calvin cycle (Benhammou et al., 2009), precursors of the synthesis of tyrosine and phenylalanine via the shikimate pathway, these amino acids are themselves precursors of the synthesis of phenolic compounds.

#### 3.3.2 Effects of fertilizers on the antioxidant and anti‐radical activities of carotenoids and phenolic compounds in pumpkins

**FIGURE 4:**
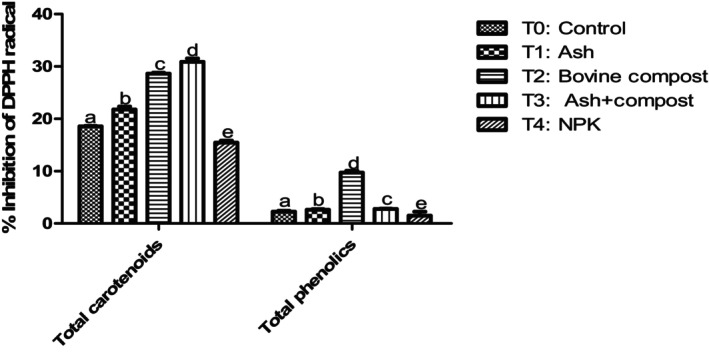
% inhibition of DPPH free radicals from terpene and phenolic extracts from pumpkin pulps. Means with the same letter in each diagram are not significantly different at the 5% level of probability using Bonferroni test.

**FIGURE 5:**
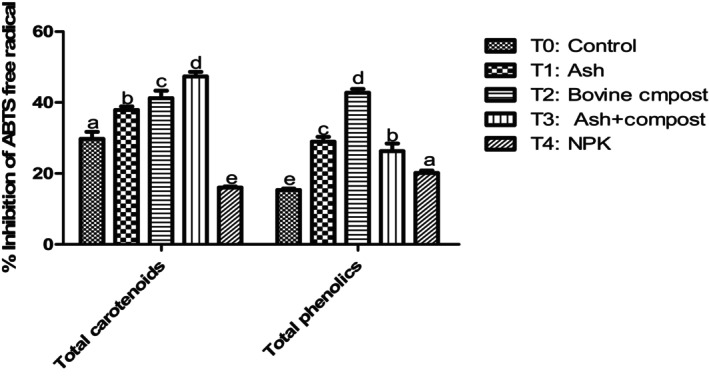
Inhibition % of ABTS free radicals from terpene and phenolic extracts from pumpkin pulps. Means with the same letter in each diagram are not significantly different at the 5% level of probability using Bonferroni test.

The antioxidant activity of total carotenoid and phenolic compound extracts was significantly influenced by fertilizers compared to negative and positive controls (Figures 4 and 5). These activities were significantly (*p* < .05) correlated to carotenoids, total phenolic, and flavonoids contents (Table 4). Compost in combination with ash (T_3_) provided squash fruits with the highest trapping power of the free radicals DPPH and ABTS. This could be explained by their carotenoids contents whose biosynthesis continues during ripening, which therefore increases the antioxidant power. Thus, the antioxidant power of the carotenoids would increase with the storage and ripening of the fruit. Table 4 shows the antioxidant activity of squash is strongly correlated with their total carotenoid, β‐carotene, and lycopene contents, with a Pearson correlation coefficient (*r*) of .98. These results could also be explained by the flavonoid content of these squash samples. In fact, flavonoids are substances with the highest antioxidant activity, and there is a positive correlation (*r*_1_ = .84 and *r*_2_ = .74) as presented in Table 4. Indeed, several authors emphasize that during the ripening process, a strong positive correlation develops between flavonoids and antioxidant activity. They conclude that ripening in fruits and vegetables is accompanied by a gradual increase in various antioxidant compounds, which is reflected in the concomitant increase in antioxidant activity (Bhandari & Lee, 2016).

**TABLE 4:** Correlation between bioactive compounds and antioxidant activity.

Coefficient de Pearson *r*	Total carotenoids	β‐Carotene	Lycopene	Total flavonoids	Total phenolics
% Inhibition DPPH	.986***	.979***	.986***	.749**	−.305^ns^
% Inhibition ABTS	.888***	.883***	.858**	.847**	−.206^ns^

*Note*: ns: non‐significant correlation at *p* < .05.

*** Significant correlation at *p* < .0001.

** Significant correlation at *p* < .001.

## 4 CONCLUSION

This research was meticulously designed to evaluate the impact of biological and chemical fertilizers on the yield, phytochemical composition – specifically carotenoids and phenolic compounds, and the antioxidant properties of Curcubita moschata. The application of bovine compost, either independently or in combination with ash, significantly enhanced the growth of squash, yielding results comparable to those obtained with the positive control NPK (20‐10‐10) fertilizer. Furthermore, the combination of ash and bovine compost as fertilizers resulted in squash fruits exhibiting the highest levels of total carotenoids, β‐carotene, lycopene, and flavonoids, along with the most potent antioxidant activity against free radicals DPPH and ABTS. This study suggests that the utilization of compost in conjunction with ash (T3) during the cultivation of squashes could markedly enhance their yield, phytochemical composition, and antioxidant potential. This finding underscores the potential of organic farming practices to promote sustainable agriculture and enhance the nutritional value of crops.

## AUTHOR CONTRIBUTIONS

**Boudjeka Guemkam Vanessa:** Conceptualization (lead); data curation (lead); formal analysis (lead); methodology (lead); validation (lead); writing – original draft (lead). **Tchiazé Ifoué Alice:** Conceptualization (equal); methodology (equal); validation (equal). **Djeukeu Asongni William:** Conceptualization (supporting); formal analysis (supporting); methodology (lead); writing – review and editing (lead). **Demasse Mawamba Adelaide:** Data curation (equal); validation (equal); visualization (equal). **Gisèle Loé‐Etame:** Conceptualization (equal); methodology (equal); supervision (equal); visualization (equal). **Nina‐Nicoleta Condurache:** Data curation (equal); methodology (equal); visualization (equal). **Ștefania‐Adelina Milea:** Methodology (equal); validation (equal); visualization (equal). **Mihaela Cotarlet:** Conceptualization (equal); supervision (equal); validation (equal); visualization (equal). **Dongho Dongmo Fabrice:** Methodology (equal); visualization (equal). **Gouado Inocent:** Conceptualization (equal); methodology (equal); supervision (equal); validation (equal). **Gabriela Iordachescu:** Methodology (equal); supervision (equal); validation (equal).

## CONFLICT OF INTEREST STATEMENT

None.

## Data Availability

The data that support the findings of this study are available from the corresponding author upon reasonable request.
